# Supplementary material for: The Dynamics of Gene Expression Unraveling the Immune Response of Macrobrachium rosenbergii Infected by Aeromonas veronii
Source: Genes (Basel). 2023 Jun 30;14(7):1383. doi: 10.3390/genes14071383 (PMC10378942; doi:10.3390/genes14071383)
Supplement: Supplementary file 1 [file genes-14-01383-s001.zip › Table S1.pdf]

Table S1

Samples	Raw reads	Clean reads	Clean bases	Q30 (%)	GC content (%)
AV6_1	22,616,031	22,263,754	6.7G	92.67	44.69
AV6_2	22,682,182	22,249,354	6.7G	92.8	41.67
AV6_3	24,166,064	23,722,890	7.1G	92.9	43.11
AV12_1	23,195,683	22,838,506	6.9G	93.01	41.75
AV12_2	23,038,504	22,630,547	6.8G	93.59	43.5
AV12_3	22,718,550	21,636,522	6.5G	93.49	43.09
AV24_1	24,040,541	23,309,585	7G	93.32	46.46
AV24_2	23,369,024	22,888,527	6.9G	94.04	46.88
AV24_3	23,686,152	23,252,948	7G	94.1	46.5
C6_1	23,283,298	22,784,336	6.8G	93.41	45.57
C6_2	24,005,222	23,655,957	7.1G	93.17	45.86
C6_3	23,593,688	23,248,847	7G	93.28	45.37
C12_1	22,782,346	22,547,508	6.8G	93.25	44.11
C12_2	22,795,828	22,565,131	6.8G	93.19	44.89
C12_3	22,248,733	21,969,078	6.6G	93.49	44.86
C24_1	23,336,887	22,926,143	6.9G	93.34	44.8
C24_2	22,338,869	21,489,762	6.4G	93.54	44.43
C24_3	23,330,073	22,930,444	6.9G	93.68	46.72

Type	Min length	Mean length	Median length	Max length	N50	N90	Total nucleotides
Transcript	301	1,716	807	28,851	3,428	614	159,764,838
Unigene	301	1,377	620	28,851	2,808	479	71,209,696
