# Supplementary material for: The Dynamics of Gene Expression Unraveling the Immune Response of Macrobrachium rosenbergii Infected by Aeromonas veronii
Source: Genes (Basel). 2023 Jun 30;14(7):1383. doi: 10.3390/genes14071383 (PMC10378942; doi:10.3390/genes14071383)
Supplement: Supplementary file 1 [file genes-14-01383-s001.zip › Table S2.pdf]

**Table S2**

| Database | NR | NT | KO | SwissProt | Pfam | GO | KOG | All |
|---|---|---|---|---|---|---|---|---|
| Number of unigenes | 17,353 | 5,130 | 7,354 | 12,481 | 16,723 | 16,720 | 6,487 | 51,707 |
| Percentage (%) | 33.56 | 9.92 | 14.22 | 24.13 | 32.34 | 32.33 | 12.54 | 100 |
