# Supplementary material for: The Dynamics of Gene Expression Unraveling the Immune Response of Macrobrachium rosenbergii Infected by Aeromonas veronii
Source: Genes (Basel). 2023 Jun 30;14(7):1383. doi: 10.3390/genes14071383 (PMC10378942; doi:10.3390/genes14071383)
Supplement: Supplementary file 1 [file genes-14-01383-s001.zip › Table S4.pdf]

Table. S4.

Pathway ID	Pathway name
ko04624	Toll and Imd signaling pathway
ko04145	Phagosome
ko04150	mTOR signaling pathway
ko04013	MAPK signaling pathway - fly
ko04611	Platelet activation
ko04062	Chemokine signaling pathway
ko04310	Wnt signaling pathway
ko04612	Antigen processing and presentation
ko04621	NOD-like receptor signaling pathway
ko04624	Toll and Imd signaling pathway
ko04670	Leukocyte transendothelial migration
ko04666	Fc gamma R-mediated phagocytosis
ko04660	T cell receptor signaling pathway
ko04350	TGF-beta signaling pathway
ko04630	Jak-STAT signaling pathway
ko04115	p53 signaling pathway
ko04620	Toll-like receptor signaling pathway
ko04657	IL-17 signaling pathway
ko04659	Th17 cell differentiation
ko04664	Fc epsilon RI signaling pathway
ko04650	Natural killer cell mediated cytotoxicity
ko04662	B cell receptor signaling pathway
ko04064	NF-kappa B signaling pathway
ko04622	RIG-I-like receptor signaling pathway
ko04623	Cytosolic DNA-sensing pathway
ko04658	Th1 and Th2 cell differentiation
ko04640	Hematopoietic cell lineage
ko04610	Complement and coagulation cascades
