# Supplementary figures and images for: The Dynamics of Gene Expression Unraveling the Immune Response of Macrobrachium rosenbergii Infected by Aeromonas veronii
Source: Genes (Basel). 2023 Jun 30;14(7):1383. doi: 10.3390/genes14071383 (PMC10378942; doi:10.3390/genes14071383)

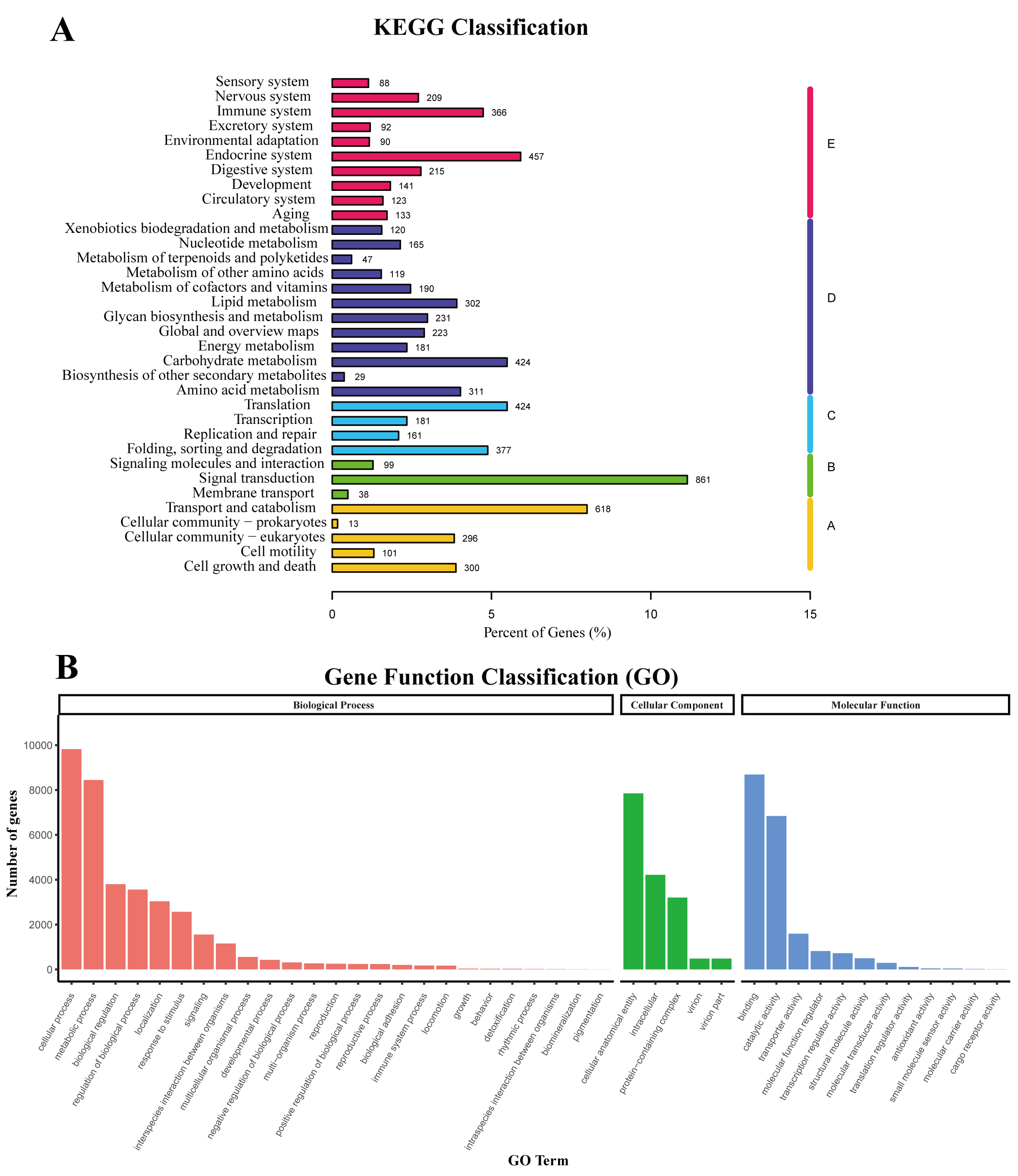

Supplement: Supplementary file 1 [file genes-14-01383-s001.zip › Figure S1.tif]
